# The Beta-1-Receptor Blocker Nebivolol Elicits Dilation of Cerebral Arteries by Reducing Smooth Muscle [Ca^2+^]_i_

**DOI:** 10.1371/journal.pone.0164010

**Published:** 2016-10-07

**Authors:** Peter Cseplo, Zoltan Vamos, Ivan Ivic, Orsolya Torok, Attila Toth, Akos Koller

**Affiliations:** 1 Institute for Translational Medicine and Szentagothai Research Centre, University of Pecs, Medical School, Pecs, Hungary; 2 Department of Central Anesthesiology and Intensive Therapy, Petz Aladar County Teaching Hospital, Gyor, Hungary; 3 Department of Anaesthesiology and Intensive Therapy, University of Pecs, Medical School, Pecs, Hungary; 4 Department of Anatomy, University of Pecs, Medical School, Pecs, Hungary; 5 Institute of Cardiology, Division of Clinical Physiology, Faculty of Medicine, University of Debrecen, Debrecen, Hungary; 6 Department of Neurosurgery, University of Pecs, Medical School, Pecs, Hungary; 7 Department of Physiology, New York Medical College, Valhalla, New York, United States of America; 8 Institute of Natural Sciences, University of Physical Education, Budapest, Hungary; Universidad Francisco de Vitoria, SPAIN

## Abstract

**Rationale:**

Nebivolol is known to have beta-1 blocker activity, but it was also suggested that it elicits relaxation of the peripheral arteries in part via release of nitric oxide (NO). However, the effect of nebivolol on the vasomotor tone of cerebral arteries is still unclear.

**Objective:**

To assess the effects of nebivolol on the diameter of isolated rat basilar arteries (BA) in control, in the presence of inhibitors of vasomotor signaling pathways of know action and hemolysed blood.

**Methods and Results:**

Vasomotor responses were measured by videomicroscopy and the intracellular Ca^2+^ by the Fura-2 AM ratiometric method. Under control conditions, nebivolol elicited a substantial dilation of the BA (from 216±22 to 394±20 μm; p<0.05) in a concentration-dependent manner (10^−7^ to 10^−4^ M). The dilatation was significantly reduced by endothelium denudation or by L-NAME (inhibitor of NO synthase) or by SQ22536 (adenylyl cyclase blocker). Dilatation of BA was also affected by beta-2 receptor blockade with butoxamine, but not by the guanylate cyclase blocker ODQ. Interestingly, beta-1 blockade by atenolol inhibited nebivolol-induced dilation. Also, the BK_Ca_ channel blocker iberiotoxin and K_Ca_ channel inhibitor TEA significantly reduced nebivolol-induced dilation. Nebivolol significantly reduced smooth muscle Ca^2+^ level, which correlated with the increases in diameters and moreover it reversed the hemolysed blood-induced constriction of BA.

**Conclusions:**

Nebivolol seems to have an important dilator effect in cerebral arteries, which is mediated via several vasomotor mechanisms, converging on the reduction of smooth muscle Ca^2+^ levels. As such, nebivolol may be effective to improve cerebral circulation in various diseased conditions, such as hemorrhage.

## Introduction

Many cerebral diseases (hypertensive encephalopathy, vascular cognitive impairment, Alzheimer’s disease, traumatic brain injury or stroke) are associated with impaired regulation of cerebral blood flow. [1]. Thus experimental and clinical investigations aim to improve regulation of cerebral blood flow by pharmacological means [2]. In addition to the improvement of the modulatory role of endothelium (for example via nitric oxide (NO) [3–6], the restoration of the appropriate regulation of smooth muscle tone of cerebral vessels is also of great importance.

One of the most frequently used therapeutic agents modulating the regulation of cardiovascular system are the so called beta blockers, among others is Nebivolol a 3^rd^ generation beta-1 blocker [7, 8]. It is a mixture of the 2 enantiomers, D-nebivolol (+SRRR) and L-nebivolol (-RSSS) [9] [8, 10]. Nebivolol possesses vasodilator properties [8, 11, 12]. Although, the primary indication of nebivolol in the treatment of hypertension [13], coronary artery disease [14], heart failure [15] and atrial fibrillation [16] is due to its beta-1 receptor mediated action, previous work by Ignarro et al [17] found that nebivolol elicits relaxation of canine coronary and pulmonary artery by stimulating endothelial NO synthesis. Supporting this conclusion, Gao et al [18] showed that nebivolol elicits endothelium-dependent relaxation in canine coronary rings, whereas further studies also showed its dilator effects on human forearm veins and arteries [19] [20].

As of today, there are no data regarding the direct effect of nebivolol on cerebral arteries without the potential brain tissues-derived confounding factors. Thus in this study we aimed to investigate the effects of nebivolol on the diameters of isolated basilar arteries isolated from rat brain, in control condition and in the presence of inhibitors of vasomotor mechanisms of know mechanisms of action in a condition, when intraluminal pressure and the environment were kept constant. During traumatic brain injury or cerebral hemorrhage the cerebral vessels are exposed to hemolysed blood, a disease condition, which prevalence is increasing substantially in world-wide [21, 22]. Previously, we have shown the extravascular hemolysed blood elicits significant constrictions of basilar arteries. Thus we also aimed to test the hypothesis that nebivolol can reverse the hemolysed blood-induced constriction of cerebral arteries [23]. The importance of the study is justified with the still limited availability of therapeutic means—without major side effects—to improve cerebral blood flow supply in diseased conditions.

## Materials and Methods

### Animals

For these experiments ~2 months-old (250±50 g) male Wistar rats (Crl:WI, Charles River Ltd., Hungary; n = 5 to 10 in each group) were used. Animals were housed on a 12h light/dark cycle and were fed ad-libitum on standard rat chow and free access to tap water. All experiments and interventions were undertaken according to the general rules and special approval of the University of Pecs Ethical Committee for the Protection of Animals in Research (BA 02/2000-8/2008), in accordance with the directives of the National Ethical Council for Animal Research and those of the EU Directive (2010/63/EU), in accordance with the ARRIVE guidelines.

### Isolation of basilar arteries (BA) and measurements of their diameter

Cerebral vessels were isolated as we previously described [23–25]. In brief, animals were anesthetized by ether narcosis and decapitated according to Institutional Animal Care and Use Committee of University of Pecs, Medical School, Pecs, Hungary. The brains were immediately removed and placed in 4°C cold Krebs’ buffer. Basilar arteries (BA) were isolated from the brain of each animal. Segments of the BAs were isolated using microsurgery instruments. Both ends of the BAs were mounted onto two glass micropipettes in a vessel chamber and pressurized to 80 mmHg with zero flow. The hydrodynamic resistances of the micropipettes were matched. Inflow and outflow pressures were controlled and measured by a pressure servo-control system (Living Systems Instrumentation, Burlington, VT, USA). Inner vascular diameter was measured with a video-micrometer system and continuously recorded using a computerized data acquisition system (LabChart 7 pro by PowerLab, ADInstruments, Australia). All vessels were allowed to stabilize for 60 min in oxygenated (21% O_2_; 5% CO_2_; 74% N_2_) superfused Krebs’ buffer (at 37°C). After the equilibration period, during which spontaneous myogenic tone developed (measured as basal diameter; BD), and the vascular responses were assessed, as reported previously [23, 26–28]. At the end of each experiment, the passive diameters (PD) of the vessels were measured at constant, 80 mmHg intraluminal pressure in the presence of Ca^2+^-free Krebs’ buffer containing the L-type Ca^2+^ channel antagonist nifedipine (10^−4^ M) to achieve maximal vasodilation.

### Assessment of endothelial function

**Fig 1:**
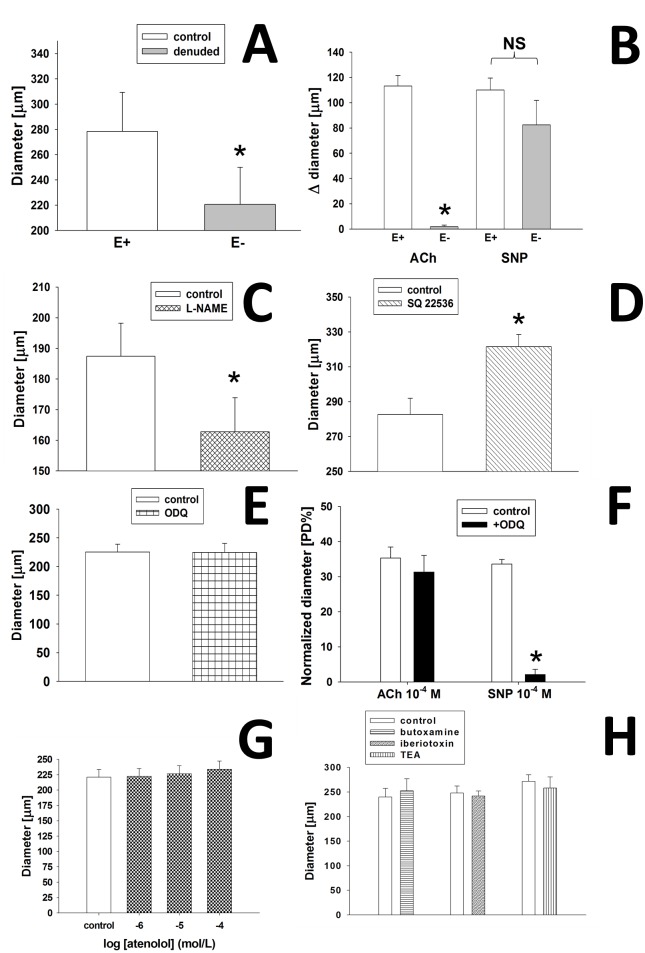
Changes in basal diameter of basilar artery (BA) in response to endothelium denudation (Fig 1(A); n = 10; E+ endothelium intact; E- endothelium denuded), NO synthase inhibitor L-NAME (Fig 1(C); n = 5), adenylyl cyclase inhibitor SQ22536 (Fig 1(D); n = 9), guanylate cyclase inhibitor ODQ (Fig 1(E); n = 12) and increasing concentration (10^−6^ to 10^−4^ M) of beta-1 adrenoceptor blocker atenolol (Fig 1(G); n = 10). To assess the vascular function acetylcholine (Ach) and sodium nitroprusside (SNP) test-doses were applied before and after the endothelial denudation (Fig 1(B); n = 10; E+ endothelium intact; E- endothelium denuded). Summary data of diameter changes (normalized diameter [PD%]) of BA in response to ACh (10^−4^ M) and SNP (10^−4^ M) in the presence of guanylate cyclase inhibitor ODQ ((Fig 1(F); n = 7). Changes in basal diameter of BA (Fig 1(H)) in response to beta-2 adrenoceptor antagonist butoxamine (BTXN; n = 5), BK_Ca_ channel blocker iberiotoxin (IBTX; n = 6), and K_Ca_ channel blocker tetraethyl-ammonium chloride (TEA; n = 10). Data are mean ± S.E.M. (*significant compared to control; p<0.05).

To evaluate the role of endothelium in the development of nebivolol induced dilation, the vascular endothelium was removed, as described previously [29]. The function of arteriolar endothelium and smooth muscle was assessed before and after endothelium denudation. Arteriolar dilations to a test dose of endothelium-dependent acetylcholine (ACh; 10^−4^ M) and endothelium independent sodium nitroprusside (SNP; 10^−4^ M) were obtained (n = 10). The endothelium was denuded by perfusing the vessel with 2 ml air (2 x [1 ml air in 5 minutes]), then filled and reperfused with Krebs’ buffer thus cleaning debris. Vessels achieved a steady-state diameter in ~15 minutes, whereupon responses to ACh and SNP were retested (Fig 1(A) and 1(B)).

### Administration of vasoactive agents and inhibitors

In the first series of experiments vasomotor function of vessels was studied in response to increased concentrations (10^−7^ M to 10^−4^ M; n = 9) of nebivolol (Berlin-Chemie/A. Menarini kft). To assess endothelial function, vascular responses to cumulative concentrations of acetylcholine (Ach; 10^−4^ M) were obtained. The intact vasomotor function of smooth muscle was verified by dilation to sodium nitroprusside (SNP; 10^−4^ M).

In separate experimental series to assess the role of nitric oxide, endothelium-mediated responses were reassessed in the presence of NO synthase inhibitor Nω-nitro-L-arginine methyl ester (L-NAME [30]; 10^−4^ M; 20 min; Koller et al, 2004; n = 5). In other experiments soluble guanylate cyclase was blocked by 1H-[1,2,4]oxadiazolo[4,3-a]quinoxalin-1-one (ODQ [31]; 10^−5^ M, 30 min, n = 12). To assess the efficacy of ODQ, acetylcholine- and SNP-induced (10^−4^ M) responses were obtained before and after incubation of vessels with ODQ. In other experimental series adenylyl cyclase was blocked by 9- (tetrahydro- 2- furanyl)- 9H- purin- 6- amine (SQ22536 [32, 33]; 10^−4^ M, 20 min, n = 9). In separate series of experiments to assess the role of beta-1 adrenoceptors in the development of nebivolol-induced dilation, we have used beta-1 adrenoceptor antagonist atenolol (10^−6^ to 10^−4^ M; n = 8). In other experiments to investigate the role of beta-2 specific adrenoceptors in the development of nebivolol-induced dilation, beta-2 specific adrenoceptor antagonist butoxamine [34, 35] was used (BTXN; 10^−4^ M, 15 min, n = 5). To assess the function of Ca^2+^-activated potassium channels in the development of nebivolol-induced dilation, Ca^2+^-activated potassium channel were blocked by tetraethylammonium [34, 35] (TEA; 10^−3^ M, 15 min; n = 10) or large conductance Ca^2+^-activated potassium channels were blocked by iberiotoxin [34, 35] (IBTX; 10^−7^ M, 15 min; n = 5). Other experimental series tested the effect of nebivolol on BA in the presence of endothelium denudation (n = 10) or specific blockers separately LNAME (n = 5), ODQ (n = 8), SQ (n = 9), BTXN (n = 5), atenolol (n = 8), TEA (n = 10) or IBTX (n = 6), respectively.

In separate series of experiment the vasomotor effect of perivascular blood was investigated by adding autologous hemolysed blood (HB) directly into the vessel chamber. Hemolysed blood (200 μL) was prepared by osmolysis from 40 μL whole blood (B) and 160 μL bidestillated water (DW) at ratio B:DW = 1:4, as previously described [23].

At the end of each experiment the passive diameters of the vessels were measured at 80 mmHg intraluminal pressure in the presence of Ca^2+^-free Krebs’ buffer containing the L-type Ca^2+^ channel inhibitor nifedipine (10^−4^ M) to achieve maximal vasodilatation. All drugs were purchased from Sigma Aldrich (Budapest, Hungary), except ODQ, SQ22536 and iberiotoxin (Cayman Europe, Tallinn, Estonia). Nebivolol was provided as gift by Berlin-Chemie/A. Menarini Ltd.

Half-maximal concentrations (EC_50_) were calculated from nonlinear regressions of the dose-response curves of nebivolol using SigmaPlot for Windows 11.0 software.

### Assessment of vascular smooth muscle calcium ion level

As described previously [23, 36, 37] changes in intracellular Ca^2+^-ion concentration were assessed with ratiometric (R) calcium-measurement at the wavelength of 340 nm and 380 nm using 5-Oxazolecarboxylic acid, 2-(6-(bis(2-((acetyloxy)methoxy)-2-oxoethyl)amino)-5-(2-(2-(bis(2-((acetyloxy)methoxy)-2-oxoethyl)amino)-5-methylphenoxy)ethoxy)-2-benzofuranyl)-, (acetyloxy) methyl ester (Fura2-AM; Invitrogen, Life Technologies, Budapest, Hungary) fluorescent dyes [38, 39]. During the incubation period of cannulated and pressurized artery the physiological Krebs solution was supplemented with Fura2-AM (5 μM) fluorescent Ca^2+^ indicator dye and BSA (bovine serum albumin; 1%) for 60 min during which spontaneous myogenic tone developed. We have used fluorescent microscope to measure intravascular Ca^2+^ concentrations by an IncyteIm2 instrument (Intracellular Imaging Inc, Cincinnati, OH, USA) by recording images (cutoff>510 nM) excited alternatively by 340 and 380 nm wavelengths. Images were recorded every 4 s and evaluated offline. Arterial Ca^2+^ concentrations were detected by calculating ratios (R) between averaged signal intensity at 340 and 380 nm excitation wavelengths in the whole arterial segment. Following the protocol of Nelson’s group the ends of the arteries were closed by pinching to prevent the Fura2 AM solution from diffusing into the vessel lumen where it could conceivably load the endothelium [40]. In addition, before loading the arteries with Fura2-AM, all the side-branches of the cerebral arteries have been closed in order to prevent the accidental endothelial loading by the fluorescent dye.

### Statistical analysis

Experimental results are presented as mean ± S.E.M. Data are expressed as either micrometer or percentage of passive diameter [PD%]. The changes in ratiometric intracellular calcium measurements are indicated as a delta ratio (ΔR). Statistical analysis was performed by one-way ANOVA (Holm-Sidak method) or Student’s t-test as appropriate by SPSS 11.0 for Windows software. P-values <0.05 were considered to be statistically significant. Figures were made by SigmaPlot 11.0 for Windows software.

## Results

### Functional assessment of the endothelium removal and the vasomotor function of endothelium and smooth muscle of isolated basilar artery

Endothelium removal reduced significantly the basal diameter from 280±30 μm to 220±29 μm (by -16±6 [PD%]; (Fig 1(A)). The presence of functional endothelium and viability of smooth muscle were evaluated by the administration of an endothelium-dependent dilator agent acetylcholine (ACh; 10^−4^ M) and a smooth muscle dependent dilator agent sodium nitroprusside (SNP; 10^−4^ M). In vessels with intact endothelium (E+), ACh elicited significant dilation (changes in diameter; ΔD: 113±8 μm; 32±5 PD%), whereas in vessels without endothelium (E-) the ACh-induced dilation was nearly completely eliminated (ΔD: 2±1 μm; 0.5±0.3 PD%). SNP elicited significant dilations both in the presence and absence of the endothelium (E+ ΔD: 110±10 μm; 32±6 PD% and E- ΔD: 83±19 μm; 26±7 PD%; Fig 1(B)). Endothelium removal was accepted when ACh-induced dilation was diminished, while SNP-induced dilation was maintained indicating the viability of smooth muscle of basilar arteries.

### Effect of nebivolol on the diameter of isolated basilar artery

**Fig 2:**
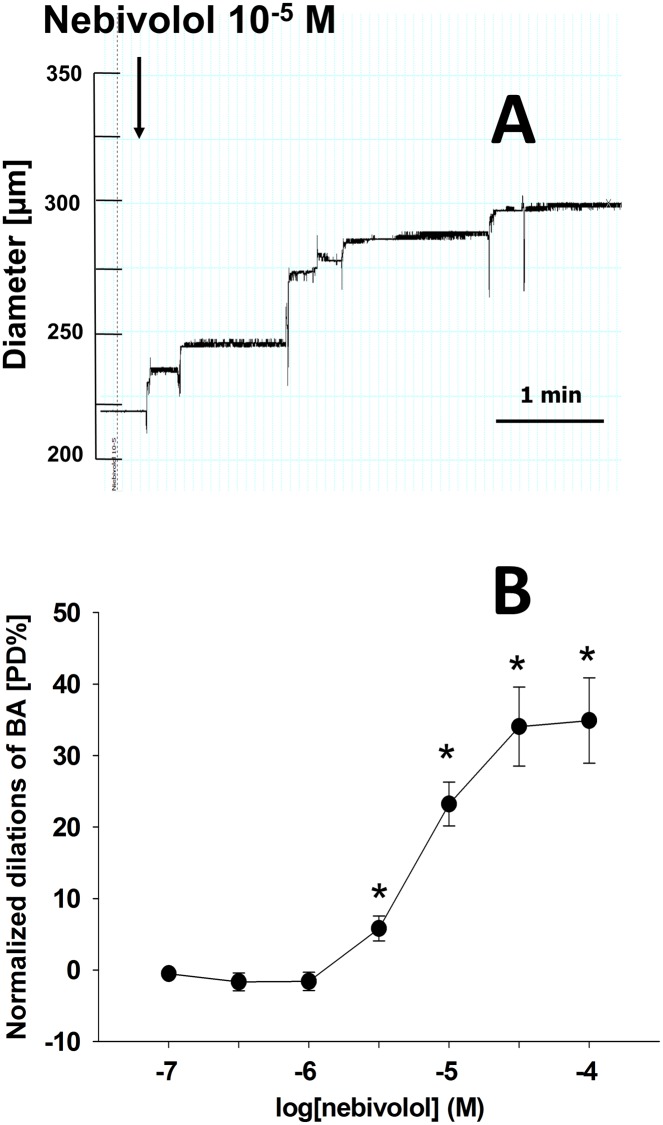
Original record of diameter changes [μm] of a rat basilar artery in response to nebivolol 10^−5^ M as a function of time (Fig 2(A)). Summary data of diameter changes (as a % of passive diameter; [PD%]) of basilar artery (BA) in response to increased concentrations of nebivolol (10^−7^–10^−4^ M). EC_50_ = 7.813 ± 0.19 x 10^−6^ M. Data are mean ± S.E.M. (*indicate significant changes compared to control; p<0.05; n = 9 (Fig 2(B)).

In the presence of 80 mmHg intraluminal pressure, the basal diameter of BA was 235±16 μm, whereas their passive diameter was 417±12 μm. Original record in Fig 2(A) shows that nebivolol (10^−5^ M) elicited an increase of the diameter of a basilar artery as a function of time (from 220 μm to 296 μm). Summary data (Fig 2(B)) shows that increasing concentrations of nebivolol elicited significant dilations of BA (from 10^−7^ M: by -0.53±0.5 [PD%] to 10^−4^ M: by 35±6 [PD%], n = 9). This finding and the EC_50_ range corresponds to those found by others [17, 34, 35], namely, that the EC_50_ of nebivolol is 7.8 ± 0.2 x 10^−6^ M. Thus we performed the experiments with specific inhibitors challenging the vasomotor effect of 10^−5^ M nebivolol.

### Effect of inhibitors with known mechanisms of action on the nebivolol-induced dilation of isolated basilar artery

Next, we have studied the effect of NOS inhibition on nebivolol-induced dilation of BA. We found that L-NAME significantly reduced the basal diameter of BA (Fig 1(C); from 188±10 μm to 162±10 μm; by -7.3±1.6 [PD%]; n = 5). The soluble guanylate cyclase blocker ODQ did not affect the basal diameter of BA (from 226±14 μm to 225±16 μm; by -0.5±2.6 [PD%]; n = 12; Fig 1(E). In control conditions, ACh and SNP elicited significant dilations of BA (35.3±3.1 [PD%] and 33.6±1.3 [PD%]). In the presence of ODQ the ACh-induced dilations were not affected significantly, while SNP-induced dilations were completely diminished of BA (31.4±4.7 [PD%] and 2.2±1.5 [PD%]; n = 7; Fig 1(F)). The adenylyl cyclase blocker SQ22536 significantly increased the basal diameter (from 283±9 μm to 322±7 μm; by 9±1.7 [PD%]; n = 9; Fig 1(D). Increasing concentration of the specific beta-1 antagonist atenolol (10^−6^ M to 10^−4^ M) did not change the basal diameter of BA (10^−6^ M: from 221±12 μm to 222±13 μm; 10^−5^ M: from 222±13 μm to 227±13 μm; 10^−4^ M: from 227±12 μm to 234±13 μm, n = 10) Fig 1(G).

Furthermore, the beta-2 adrenoceptor antagonist butoxamine (BTXN) did not change the basal diameter (control 240±17 μm, BTXN: 252±25 μm, n = 5; Fig 1(H). Similarly, the K_Ca_ channel blocker tetraethylammonium (TEA) (control: 272±14 μm, TEA: 258±23 μm, n = 10) or the BK_Ca_ channel antagonist iberiotoxin (IBTX) (control: 248±15 μm, IBTX: 242±10 μm, n = 6; Fig 1(H)) did not affect the basal diameter of basilar arteries, confirming the results of previous studies [35].

**Fig 3:**
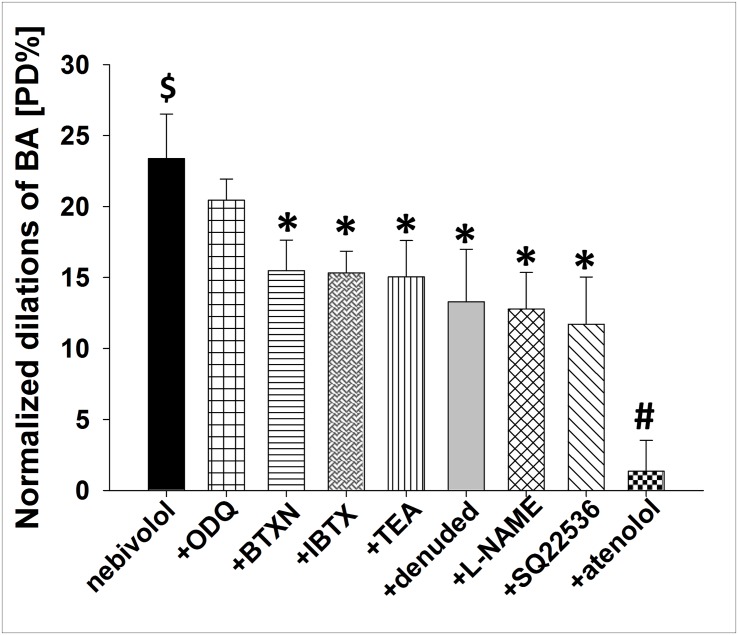
Summary data of diameter changes (normalized dilation; % of passive diameter; [PD%]) of BA in response to nebivolol (10^−5^ M) in the presence of guanylate cyclase inhibitor ODQ, beta-2 selective adrenoceptor antagonist butoxamine (BTXN), large conductance calcium-activated potassium channel antagonist iberiotoxin (IBTX), K_Ca_ channel antagonist tetraethyl-ammonium chloride (TEA),endothelium denudation, NO synthase inhibitor L-NAME, adenylyl cyclase inhibitor SQ22536 and beta-1 selective adrenoceptor antagonist atenolol. Data are mean ± S.E.M. (**$** indicate significant change compared to basal diameter, p<0.05; * indicate significant changes compared to nebivolol, p<0.05; **#** indicate significant change between nebivolol vs atenolol, p<0.05).

Summary data (Fig 3) shows that nebivolol (10^−5^ M) elicited significant dilations of BA (by 23.4±3 [PD%]; p<0.05; n = 9). Nebivolol-induced dilation of BA was not affected after incubation and in presence of ODQ (by 20.5±1.5 [PD%]; p>0.05; n = 8).

The reduction of nebivolol-induced dilation increased with the following order: butoxamine (by 15.5±2.1 [PD%]; p<0.05; n = 5), iberiotoxin (by 15.3±1.5 [PD%]; p<0.05; n = 6), TEA (by 15.04±2.55 [PD%]; p<0.05; n = 10), endothelium-denudation (by 13.3±3.7 [PD%]; p<0.05; n = 10), L-NAME (by 12.8±2.6 [PD%]; p<0.05; n = 5), SQ22536 (by 11.7±3.3 [PD%]; p<0.05; n = 9). Whereas, atenolol (by 1.4±2.2 [PD%]; p<0.05; n = 8), completely eliminated the dilation to nebivolol.

### Changes in [Ca^2+^]_i_ of isolated basilar artery in response to nebivolol

**Fig 4:**
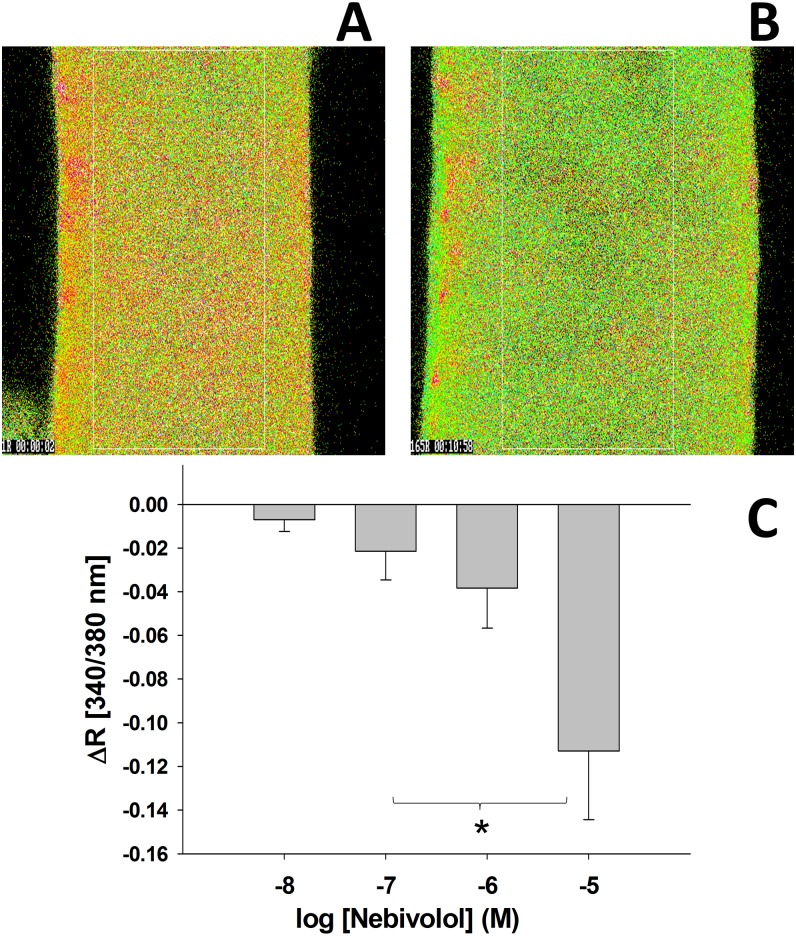
Representative pictures of Fura-2 AM fluorescence of ex vivo preparation of BA in absence (control; Fig 4(A)) and presence (Nebi; Fig 4B)) of nebivolol (10^−5^ M). Green color indicates low vascular Ca^2+^ level and red color indicates high vascular Ca^2+^ level. **S**ummary data of changes in ratiometric signal (delta R) indicates decreasing vascular [Ca^2+^]_i_ of BA in response to increasing concentrations of nebivolol (10^−8^–10^−5^ M). Data are mean ± S.E.M. *indicate significant changes between nebivolol 10^−7^ M and 10^−5^ M; p<0.05); n = 9 (Fig 4(C)).

Original images (Fig 4(A) and 4(B)) show that nebivolol elicited significant reduction (green color indicate low and red color indicates high vascular Ca^2+^ levels) in ratiometric Ca^2+^-signal (delta Ratio; ΔR) and elicited an increase in diameter of a basilar artery. Summary data (Fig 4(C)) shows that nebivolol decreased the ratiometric Ca^2+^ signal (ΔR) in a concentration-dependent manner (10^−8^ M: -0.007±0.005 vs. 10^−5^ M: -0.113±0.031). Nebivolol elicited dilations of basilar arteries with the consequent decrease in intracellular Ca^2+^ concentration.

### Effect of nebivolol on the diameter of isolated basilar artery in the presence of hemolysed blood

**Fig 5:**
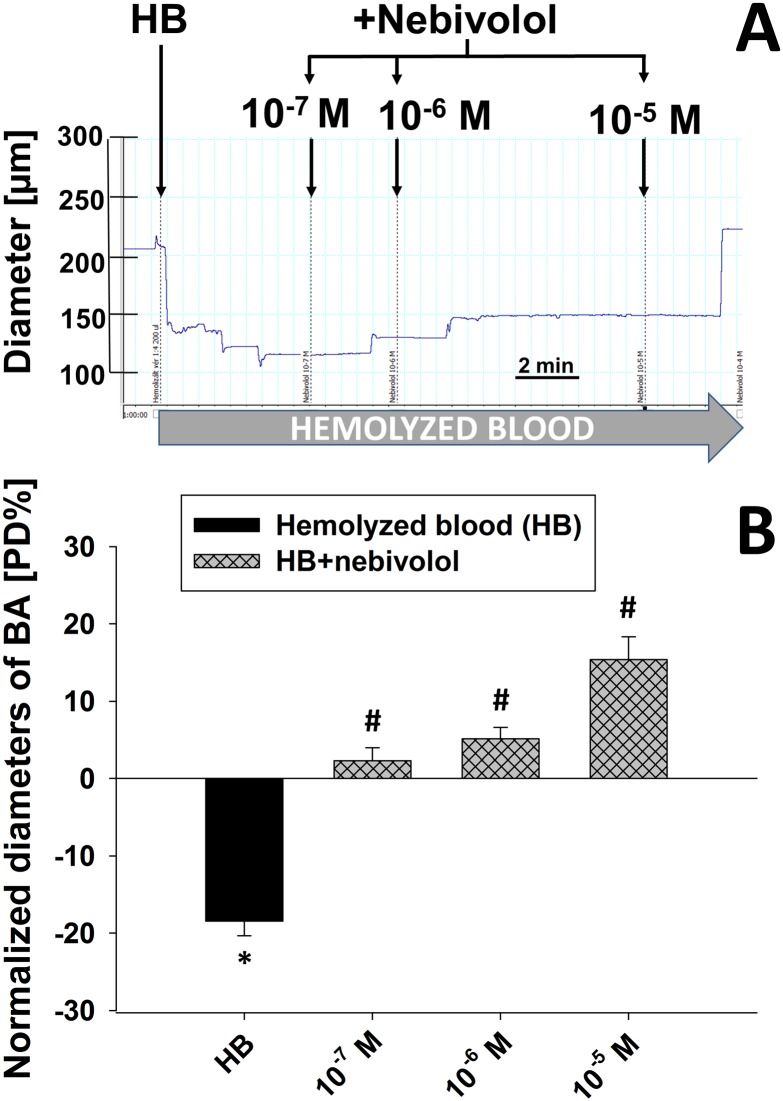
Original record shows the vasomotor function of perivascular hemolyzed blood (HB) and the vasomotor function of additional increased concentrations of nebivolol (from 10^−7^ M to 10^−5^ M) of basilar artery. The diameter was not followed continuously in Fig 5(A). Summary data of changes in normalized diameter (% of passive diameter; [PD%]) of basilar arteries in response to hemolyzed blood (HB) and in response to nebivolol (from 10^−7^ to 10^−5^ M) in the presence of HB; n = 6. Data are mean ± S.E.M. (* indicate significant difference compared to basal diameter, p<0.05; # indicate significant difference between HB and nebivolol in the presence of HB, p<0.05); Fig 5(B).

In a recent study we have found that perivascular hemolysed blood induces substantial constriction of isolated basilar arteries by increasing smooth muscle [Ca^2+^]_i_ [23]. Original record in Fig 5(A) shows that adding hemolyzed blood to the perivascular area elicits substantial constriction of basilar arteries (BA, from 210 μm to 115 μm). Then, additional administration of increasing concentrations of nebivolol into the vessel chamber elicited dilation in a concentration-dependent manner (10^−7^ M: 125 μm; 10^−6^ M: 148 μm; 10^−5^ M: 224 μm) and in essence, completely reversed the constrictor effect of HB. In Fig 5(B) summary data shows that the constrictor effect of perivascular hemolyzed blood could be reversed by adding nebivolol in the presence of HB. HB caused significant constrictions of BA (from 219±11 μm to 148±11 μm; -18,5±1,9 [PD%]), which was completely reversed by adding increasing concentrations (from 10^−7^ M to 10^−5^ M) of nebivolol (10^−7^ M: 2,3±1,6 [PD%]; 10^−6^ M: 5,1±1,4 [PD%]; 10^−5^ M: 16±3 [PD%]). Interestingly, nebivolol induced dilation at a concentration of 10^−5^ M reached the control basal diameter of basilar arteries (control basal diameter 219±11 μm; HB: 148±11 μm; Nebivolol 10^−5^ M in the presence of HB: 209±9 μm).

## Discussion

As far as we know, this is the first study to demonstrate that nebivolol elicits dilation of isolated cerebral arteries. Nebivolol seems to have specific regional effect, because the cerebrovascular dilation is mediated by several, parallel acting vasomotor mechanisms including endothelium-derived NO and cAMP linked mechanisms, beta-1/2 adrenoceptors, hyperpolarizing factor(s)/ BK_Ca_ channels, finally, converging on the reduction of smooth muscle Ca^2+^ levels. In addition, nebivolol reversed the hemolysed blood-induced constriction. Thus nebivolol may be effective in the improvement of cerebral circulation in diseased conditions.

Interestingly, on the basis of early investigations and clinical applications—for many years—only the beta-1 receptor effect of nebivolol was considered as a potential therapeutic value. Then, first Ignarro et al showed that nebivolol has additional effect, namely eliciting dilation of peripheral arterial vessels, primarily, via activating the NO-pathway [3]. Previous findings [4–6, 41] initiated our study in cerebral arteries, in which the potential, additional vasomotor effects of nebivolol could be elucidated. The results of our studies show that nebivolol dilates cerebral arteries, an effect which is—in part—independent of beta-1 receptor mediation, suggesting—at least the possibility—that *in vivo* it can facilitate cerebral blood flow. The findings that dilator effect of the cerebral arteries is mediated by reduction of smooth muscle Ca^2+^ also suggest that even in the presence of endothelial dysfunction nebivolol can elicit dilation. These assumptions, of course should be confirmed by future studies.

### Vasomotor effects of Nebivolol

Nebivolol is a 3^rd^ generation widely used beta-blocker [7–10] drug with antiarrhythmic and antihypertensive effect [8, 42]. In addition, it has been discovered that nebivolol has vasomotor effects, which seems to unrelated to its direct beta-1 receptor mediated action. Namely in peripheral arteries nebivolol induces dilation [8, 11] in part, via increasing the activity of eNOS (endothelial nitric oxide synthase) thereby upregulating NO-cGMP (cyclic-guanylate monophosphate) pathway [17] resulting in vasodilation [19, 20].

It seems that the effects of nebivolol are organ/tissue specific, because in rat aortic rings Ignarro [17] found both endothelium-dependent and -independent mediation of relaxations, but several other mechanisms have been described underlying vasodilator effect of nebivolol. For example Evangelista et al. reported that antioxidant properties of nebivolol can increase the “surviving” level of NO by reducing its oxidative inactivation in human umbilical vein endothelial cells, and that beta-1/2/3 adrenoceptors may play role in the development of nebivolol-induced dilation [43]. Moreover, Georgescu et al. showed nebivolol induces beta-2 adrenoceptor-mediated and Ca^2+^ activated potassium channel induced hyperpolarization with the consequent relaxation in mice renal arteries [35], whereas, Tran-Quang et al. has demonstrated nebivolol-induced specific, either beta-2 and beta-3 agonist or alpha-1 and beta-1 antagonist adrenoceptor-mediated relaxations in rat aortic rings [44]. It was also observed that nebivolol elicits the release of hyperpolarizing factor via activation of calcium activated potassium channels [34] that—in part—maintains vessel relaxation when eNOS (endothelial NO synthase) is inhibited [17]. Such parallel and backup mechanisms would be particularly important, when NOS (NO synthase) activity, NO bioavailability, and/or other signaling mechanisms are impaired, such as increased oxidative stress and ischemic heart diseases [45–47].

Interestingly, several studies reported that other beta-blockers also induce relaxations in both peripheral and central arteries. Among others, Sakanashi et al. found propranolol-induced relaxation in canine coronary arteries and raised the possibility that propranolol reduces the calcium-influx [48]. Priviero et al showed that propranolol-induced relaxation in rat aorta and mesenteric artery may occur, independent of beta-adrenoceptor blockade, via inhibition of calcium influx [49]. Similarly, Cekic et al suggested, that nonspecific beta-blocker propranolol exhibited calcium antagonist activity in rat basilar arteries [50].

The finding of these studies lead us to hypothesize that nebivolol elicit relaxation of cerebral arteries, an idea which has not yet been tested in a controlled experimental conditions, but can have important clinical significance.

### Nebivolol induces dilation of cerebral arteries

Interestingly, as mentioned above, there are no data available regarding the vasomotor effects of nebivolol in cerebral vessels. Thus we aimed to characterize the dilator properties of nebivolol in isolated basilar arteries, in such conditions, in which the intraluminal pressure and extravascular environment were controlled. Based on previous studies [17, 34, 35, 44] we have used nebivolol in a range of 10^−7^ to 10^−4^ M which is in accordance with a range of concentrations used by others. Our data show significant dilations of BA in response to concentration-dependent administration of nebivolol (Fig 2). Since the EC_50_ is 7.8 ± 0.19 x 10^−6^ M, we performed measurements with specific inhibitors near this EC_50_ range in the presence of nebivolol arbitrarily at 10^−5^ M. Despite the calculated pharmacologically relevant plasma concentrations in humans after taking 5 mg nebivolol orally, it is in the nM range, but it still elicits a significant decrease in systemic vascular resistance [44], and it may result in dilation in human cerebral arteries. It is also possible that smaller cerebral vessels are even more sensitivity to nebivolol, as it is a general characteristics of microvessels that their sensitivity to various drugs and stimuli increases as the size (diameter) of vessels decreases [51].

### Role of endothelium in nebivolol-induced dilation of basilar artery

The basilar artery (BA) branching of from vertebral arteries thus it has a special role in maintaining an adequate blood flow and blood pressure, which then equilibrate in the Circle of Willis and brainstem, as well [52].

It is well known endothelium is an important active mechanical and biological interface between the circulating blood and surrounding tissues. It has many special function from gas exchange to vasomotor [53] and barrier function. Vasomotor function of endothelium includes sensing of wall shear stress associated with blood flow velocity changes and other autocrine and paracrine signal transductions mechanism and substances [54] [55]. Thus in many instances, removal or impairment of endothelium has a significant influence on vasomotor tone and function. In the present study we have confirmed previous findings that absence of endothelium significantly decreased the basal diameter of cerebral arteries by ~ 60 μm (Fig 1(A)) supporting a putative role for maintaining basal tone [1, 56, 57].

### Role of NO in maintaining basal tone of BA

Results coming from aforementioned investigations demonstrated a special role of NO in maintaining basal tone. As previously described [4], we have also confirmed that in basilar arteries endogenous NO (produced by endothelium) contributes to the development of basal vascular tone as administration of L-NAME significantly decreased (Fig 1(C)) the basal diameter by ~ 30 μm, supporting previous findings [24, 52], and underlying the physiological importance of NO in the regulation of vasomotor tone of cerebral arteries [1, 56, 57].

### Endothelium dependent and smooth muscle dependent vasomotor effects of nebivolol

Importantly, nebivolol was able to induce significant, but reduced dilation of basilar artery in absence of endothelium (Fig 3), suggesting that nebivolol may act—in part—by mediation of vascular endothelium, but it has direct smooth muscle effects as well. The findings of our experiments are in accordance with those of previous studies showing that nebivolol-induced relaxations are significantly greater in the presence of intact endothelium than in the absence of endothelium. [12, 17–20, 35, 42].

### Role of eNOS-NO and cGMP/cAMP pathways in the development of nebivolol-induced dilation

Previous studies showed that the basal tone of peripheral arteries is modulated by NO [3, 29, 58] and that nebivolol induces an eNOS/NO-mediated dilations in peripheral arteries [17–20, 42]. In the recent study we have found that presence of eNOS blocker L-NAME significantly decreased the basal diameter of BA in accordance with previous studies [4–6, 41] (Fig 1(C)). More importantly, we found that the dilations of isolated rat basilar arteries to nebivolol was also reduced in the presence of L-NAME (Fig 3), suggesting that NO is involved in the mediation of the response. Most previous studies suggest that NO increases the level of the smooth muscle soluble guanylate cyclase (sGC) enzyme producing cyclic-guanylate monophosphate (cGMP). Then, cGMP activates protein kinase-G (PKG) [59], which is responsible for inactivating the myosin light chain kinase (MLCK). MLCK would be the essential enzyme in the development of vasoconstriction, which is inhibited by elevated level of PKG thus resulting in dilation [60].

**Fig 6:**
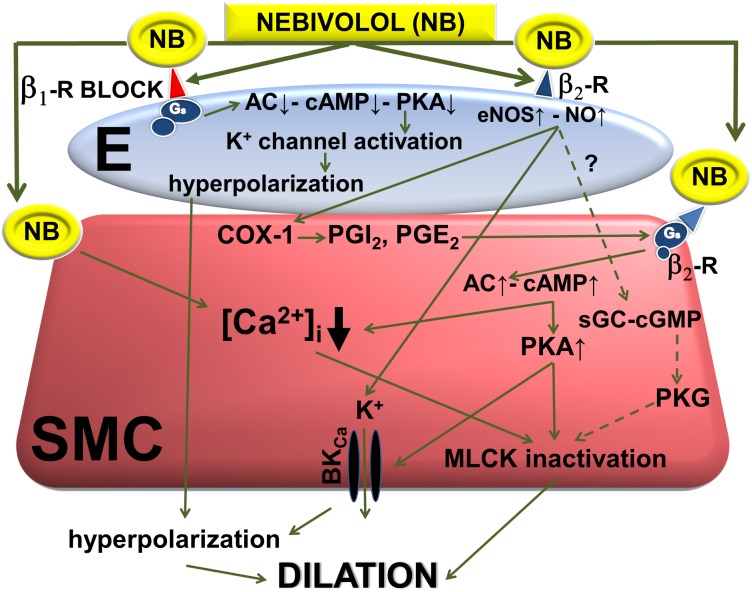
Proposed mechanisms of action of nebivolol (NB)-induced dilation of cerebral arteries (E, endothelium; SMC, vascular smooth muscle cell). These findings demonstrate that: 1) in isolated cerebral arteries nebivolol elicits significant dilations, 2) which may be—in part—due to beta-2 adrenoceptor (B2-R) mediated, endothelium-dependent NO and cAMP mechanisms resulting in either reduced [Ca^2+^]_i_, and smooth muscle hyperpolarization. 3) On the other hand, its action seems to be mediated—in part—by beta-1 (B1-R) specific blocking ability connected with parallel induced vasodilation with endothelium derived hyperpolarization. 4) contribution of cGMP and other ion channels seem to be less important. These findings can contribute to a better understanding of the complex effects of this beta-1-receptor blocker on cerebral circulation and implicate important novel therapeutic potentials to improve cerebral blood flow in diseased conditions.

In addition, previous studies assigned an important role for the cyclic-adenosine monophosphate (cAMP) produced by adenylyl cyclase (AC) mechanism in mediation of various dilator responses of vessels [61]. cAMP can 1) activate protein kinase-A (PKA) thereby inactivating MLCK resulting in dilation (Fig 6), 2) activate calcium ATPase thus reducing [Ca^2+^]_i_ leading to vasodilation, 3) inhibit calcium-calmodulin complexes thereby inducing vasodilation. Therefore we performed experiments to block synthesis of both cAMP [32] (with SQ22536) and cGMP (with ODQ).

In contrast to previous studies (canine coronary and pulmonary arterial rings, human forearm) [17–20], but similarly to Ogawa et al. [62], we have found that the sGC-inhibitor ODQ did not decrease the basal diameter (Fig 1(E), and did not reduce the nebivolol-induced dilations (Fig 3) suggesting that intracellular sGC/cGMP pathway does not contribute significantly to the development of nebivolol-induced dilations of cerebral vessels. We explain these findings by the presence of sGC/cGMP independent NO pathway(s) [62–69]. Indeed, there is evidence that in certain vessels the vasodilator effect of NO are mediated via COX-dependent, cAMP mediated pathway [62, 67–69], for example in rat retinal blood vessels [62, 63] that are ontogenetically are closely related to cerebral vessels. However, it has also been shown that NO can act directly to Ca^2+^-dependent K^+^ channels in vascular smooth muscle cells, which cause a dilator response [64, 66] or by decreasing Ca sensitivity of arteriolar smooth muscle [37].

Our data shows that AC inhibitor SQ22536 exhibited significant increase in basal diameter (Fig 1(D)) suggesting the presence of a tonic release of a constrictor factor and/or SQ22536 may elicit nonspecific enzyme inhibition [70]. Yarova et al. showed that beta-1 agonist in presence of endogenous/exogenous inductor (acetylcholine) suppresses vasodilation via increase in endothelial cAMP level [71]. If endothelial AC is blocked it can mimic the beta-1 antagonist induced vasodilation, supporting the findings that dilation was observed in the presence of SQ22536. In the presence of SQ22536 nebivolol-induced dilation was significantly reduced (Fig 3), suggesting the involvement of an AC/cAMP, rather than a sGC/cGMP mechanism in the development of nebivolol-induced dilation of basilar arteries, which seems to be activated by NO. These results are in agreement with findings of others [62], which showed that in certain vessels NO acts via the cAMP pathway, including COX1-PGI_2_/PGE_2_-Gs pathways [62, 63, 67–69]. Thus we propose that cerebrovascular dilator effects of nebivolol depend—in part—on endothelial mechanisms and the eNOS/NO-AC/cAMP pathway.

### Involvement of beta-adrenoceptor and BK_Ca_ channels in the development of nebivolol-induced dilation of BA

Cekic et al. showed the specific beta-1 adrenerg antagonist atenolol did not elicit relaxation of rat cerebral arteries, whereas the non-selective propranolol induced relaxation [50]. Tran-Quang et al demonstrated beta-1 receptor antagonist did not affect significantly the nebivolol-induced relaxation in rat aortic rings, suggesting beta-1 receptors do not contribute to the development of nebivolol-induced dilation [44]. Interestingly, Yarova et al showed on rat mesenteric arteries that endothelial beta- adrenoceptor agonists decreased ACh-induced dilation, whereas in the presence of beta-1 antagonist atenolol, ACh-induced dilation remained intact [71]. These findings raise the possibility that specific beta-1 adrenoceptor antagonist—in certain circumstances—may have the characteristics of functional agonist, via blocking endothelial Gs-AC-cAMP-PKA pathway, thereby decreasing the inactivation of Ca-dependent potassium channels, resulting in endothelial and smooth muscle hyperpolarization (through myoepithelial gap junction), leading to vasodilation [71].

In the present study we show that beta-1 selective antagonist atenolol did not significantly affect basal diameter of BA (Fig 1(G), which may be due to either absence of an endothelial inductor factor [50], or it may have parallel beta-2 receptor antagonist properties, as Nuttall reported [72]. Furthermore we found that atenolol almost completely eliminated the dilations to nebivolol (Fig 3), which can be explained by either the presence of a beta-2 antagonist effect of atenolol (resulting in inhibition of dilation) [72] or by occupying beta-1 adrenoceptors (competitive antagonism), when nebivolol could act in presence of NO. Accordingly, one can suggest that nebivolol-induced dilation is attributed—in part—via beta-1 adrenoceptors.

Studies of Georgescu et al. [35], suggest that beta-2 adrenoceptors may play important role in the development of nebivolol-induced dilation in mice renal artery. This prompted us to investigate the potential mediating role of beta-2 adrenoceptors in the vasomotor action of nebivolol in basilar artery. Fig 3 shows that presence of beta-2 adrenoceptor antagonist butoxamine (BTXN) significantly decreased the nebivolol-induced dilations of BA. Beta-2 receptors may be expressed both on endothelial cell and on vascular smooth muscle cell. The endothelium-dependent, NO mediated pathway may be one of the major functions of beta-2 receptors. Stimulation of beta-2 receptors (expressed on vascular smooth muscle cells) leads to the activation of AC-cAMP-PKA pathway, which induces dilation—in part—by inactivating MLCK and partly by activating BK_Ca_ channels [73] and hyperpolarization (Fig 6).

Bolotina et al demonstrated the involvement of BK_Ca_ channels in NO-mediated vasodilation [64] and Georgescu et al showed the role of BK_Ca_ channels in the development of nebivolol-induced dilation [34]. In line with previous findings, our results showed that the BK_Ca_ channel antagonist iberiotoxin [IBTX] and TEA significantly reduced nebivolol-induced dilation (Fig 3), suggesting an important role of BK_Ca_ and hyperpolarization, mediated by endothelial NO, and cAMP-PKA, and via beta-1 and beta-2 receptor mediated pathways, which seem to be specific for cerebral arteries.

### Nebivolol decreases [Ca^2+^]_i_ in basilar arteries

Previous studies also suggested that therapeutic effects of beta-blockers could be attributed to endothelium-independent mechanisms [48–50]. Sakanashi et al. found propranolol-induced relaxation in canine coronary arteries and raised the possibility that propranolol reduces the calcium-influx [48]. Priviero et al showed that propranolol-induced relaxation in rat aorta and mesenteric artery may occur, independent of beta-adrenoceptor blockade, via inhibition of calcium influx [49]. Similarly, Cekic et al suggested, that nonspecific beta-blocker propranolol exhibited calcium antagonist activity in rat basilar arteries [50].

Since we have found that nebivolol elicits dilation of basilar arteries, we hypothesized that regardless of proximal pathways, nebivolol reduces the Ca^2+^ level in smooth muscle, which then results in dilation. Thus we investigated parallel changes in the vascular Ca^2+^-signal (R) and the diameter in isolated basilar arteries utilizing the ratiometric method used in our previous studies and others [23, 36, 38, 39]. The data of present study show that increasing concentrations of nebivolol caused significant decrease in vascular Ca^2+^-signal (R) (Fig 4), indicating decrease in vascular [Ca^2+^]_i_ concentration. The decrease in ratiometric Ca^2+^ signal and the consequent dilation suggest that the signal is coming primarily from the vascular smooth muscle layer. This is congruent with our functional measurements of diameter changes and suggests that the final signaling mechanism by which nebivolol elicits dilation of cerebral arteries is the reduction of smooth muscle intracellular Ca^2+^ concentration.

### Clinical implications

Searching for effective pharmaceutical treatments to improve cerebral blood flow in diseased conditions or during aging is an ongoing effort in clinical practice. In ischemic condition, such as transient ischemic effect [74] or stroke [ischemic [75], hemorrhagic [76] the resistance of cerebral vessel greatly increases inhibiting the appropriate supply of brain with blood flow. Our findings show sizeable dilations of basilar cerebral arteries to nebivolol in the absence of neural or other tissue factors. Thus, we can speculate that nebivolol could be used in clinical settings to improve cerebral blood flow, even in diseased conditions.

### Reversal of hemolysed blood-induced constriction of isolated BA

In recent study we have found that perivascular hemolysed blood induces substantial constriction of isolated basilar arteries by increasing smooth muscle [Ca^2+^]_i_ [23]. Thereby we have tested the dilator effect of nebivolol in the presence of perivascular hemolyzed blood adding the chamber solution. Interestingly, we have found that hemolyzed blood (HB)-induced constriction of cerebral arteries could be reversed by adding increasing concentrations of nebivolol into the vessels chamber. This finding strongly suggests that nebivolol have potential therapeutic value, for example, in patients with subarachnoid hemorrhage-induced cerebrovascular spasm.

Extrapolating these experimental findings to clinical area, may open up novel therapeutic possibilities for this novel 3rd generation beta-1 blocker. A previous trial (BEST) using propranolol to assess its effects on cerebral function in patients with subarachnoid hemorrhage and those suffering from acute stroke showed promising improvement in the long range, but more early death [77]. Nebivolol has been shown to be very safe and effective beta-1-blocker [78] and is used in much lower concentration than propranolol [70], thus one can assume that it may have less side effects in transient ischemic attack (TIA) or in various stroke conditions. Nebivolol seems to be an appropriate antihypertensive medication in subarachnoid hemorrhage patients following endovascular treatment of bleeding cerebral aneurysms [79]. Thus by dilating cerebral arteries, nebivolol may be useful in stroke patients restoring cerebral perfusion under lower systemic blood pressure. Future clinical studies are needed to elucidate this possibility and document the beneficial effects of nebivolol on cerebral circulation in various diseased conditions.

### Limitation of the Study

To facilitate comparison regarding mediation of the vasomotor responses to nebivolol we have followed the experimental protocols of Georgescu et al [34, 35]. Accordingly, we have only used the aforementioned specific K_Ca_ channel blockers on cerebral arteries. Furthermore, because previously we have tested both TEA and iberiotoxin [80–83] we felt that we used suitable experimental protocols. Although, it may be viewed as a limitation of our study, it is also likely that combined application of inhibitors may exert unexpected and unknown effects that would be difficult to explain or interpret. Nevertheless, in future studies one would make an attempt to block IK_Ca_ channels (TRAM34) and SK_Ca_ channels (Apamin) individually and simultaneously with appropriate controls.

## Conclusions

This is the first study showing that nebivolol induces substantial dilation of cerebral arteries and revealed the specific potential intracellular signaling mechanisms. The main novel findings are that in isolated rat cerebral arteries nebivolol elicits significant dilations, which is mediated by several parallel intracellular parallel pathways, including endothelium-derived NO and cAMP linked mechanisms, beta-1/2 adrenoceptors, hyperpolarizing factor(s)/ BK_Ca_ channels, that are all seem to converge on the reduction of [Ca^2+^]_i_, level. In addition, nebivolol reversed the constrictor effect of hemolysed blood, which was shown earlier to increase smooth muscle Ca^2+^.

These findings can contribute to a better understanding of the complex effects of this beta-1-receptor blocker on cerebral circulation and implicate important novel therapeutic potentials to improve cerebral blood flow in diseased conditions, such as aging and hemorrhage.

## Abbreviations

AC: adenylyl cyclase
BA: basilar artery
BK_Ca_: large conductance calcium-activated potassium channel
BTXN: butoxamine
cAMP: cyclic adenosine monophosphate
cGMP: cyclic guanosine monophosphate
eNOS: endothelial NO synthase
Fura2-AM: 5-Oxazolecarboxylic acid, 2-(6-(bis(2-((acetyloxy)methoxy)-2- oxoethyl)amino)-5-(2-(2-(bis(2-((acetyloxy)methoxy)-2-oxoethyl)amino)-5-methylphenoxy)ethoxy)-2-benzofuranyl)-, (acetyloxy) methyl ester
IBTX: iberiotoxin
K_Ca_: Calcium-activated potassium channel
L-NAME: Nω-nitro-L-arginine methyl ester
NO: nitric oxide
ODQ: 1H-[1,2,4]oxadiazolo[4,3-a]quinoxalin-1-one
sGC: soluble guanylate cyclase
SQ22536: 9- (tetrahydro- 2- furanyl)- 9H- purin- 6- amine
TEA: tetraethyl ammonium chloride
PD: passive diameter
